# Supplementary material for: Volatiles of All Native Juniperus Species Growing in Greece—Antimicrobial Properties
Source: Foods. 2023 Sep 20;12(18):3506. doi: 10.3390/foods12183506 (PMC10530231; doi:10.3390/foods12183506)
Supplement: Supplementary file 1 [file foods-12-03506-s001.zip › foods-2609909-supplementary.pdf]

SUPPLEMENTARY FILE

Volatiles of all native *Juniperus* species growing in Greece-Biological properties

Evgenia Fotiadou[1], Evgenia Panou[1], Konstantia Graikou[1], Fanourios-Nikolaos Sakellarakis[2], Ioanna Chinou[1,*]

[1] Laboratory of Pharmacognosy and Chemistry of Natural Products, Faculty of Pharmacy, National and Kapodistrian University of Athens, Panepistimiopolis, Zografou, 15771, Athens, Greece; eugenia.fot@gmail.com (E.F.); evgenia.panou39@gmail.com (E.P.); kgraikou@pharm.uoa.gr (K.G.); ichinou@pharm.uoa.gr (I.C.)

[2] Society for the Protection of Prespa, Agios Germanos, 53077, Florina, Greece; fansakell@gmail.com (F-N.S.)

* Correspondence: ichinou@pharm.uoa.gr;

Figure S1: Map with the collection areas of Greek Juniperus samples

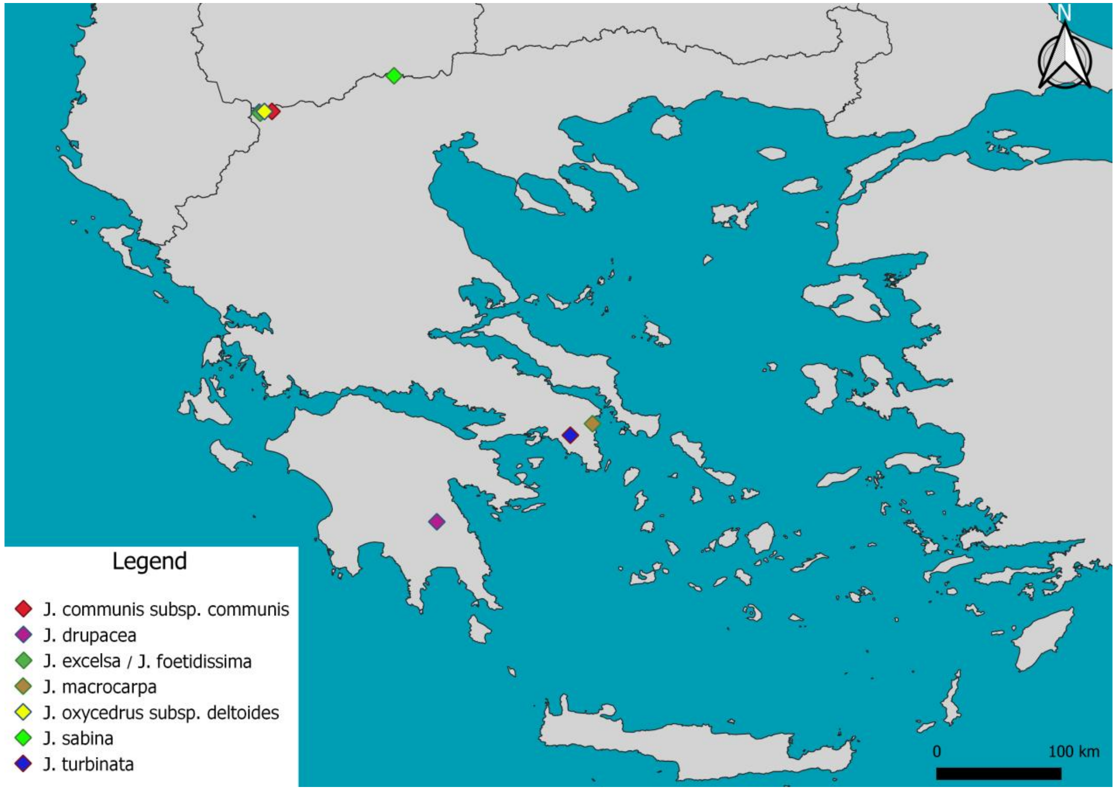

Table S1: Chemical composition of the Juniperus spp. essential oils from GC-MS analysis.

		KI	*Je-leaves*	*Je-cones*	*Jf-leaves*	*Jf-cones*	*Jcc-leaves*	*Jcc-cones*	*Jm-leaves*	*Jm-cones*	*Jt-leaves*	*Jt-cones*	*Jod-aerial*	*Jd-leaves*	*Jd-cones*	*Js-leaves*	*Js-cones*
1.	α-Pinene	939	12.36	30.59	15.65	36.40	15.29	22.50	11.11	12.07	26.55	43.11	7.36	5.72	25.8	6.35	13.12
2.	Camphene	955	nq	1.44	nq	nq	nq	nq	nq	nq	1.72	0.89	nq	nq	nq	nq	nq
3.	Thuja-2,4(10)-diene	956	nq	nq	nq	nq	nq	nq	nq	nq	nq	0.41	0.79	nq	nq	nq	nq
4.	Verbenene	963	nq	nq	nq	nq	nq	nq	nq	nq	nq	0.82	nq	nq	nq	nq	nq
5.	Sabinene	977	6.53	nq	nq	nq	13.50	10.12	nq	nq	nq	nq	nq	nq	nq	36.13	36.87
6.	β-Pinene	980	nq	2.84	0.58	2.38	nq	nq	0.20	0.77	1.55	5.12	0.38	nq	1.5	nq	nq
7.	Myrcene	993	1.67	3.67	1.34	3.26	3.15	5.05	0.46	1.28	3.14	4.02	3.15	1.79	2.86	4.38	4.33
8.	α-Phellandrene	1005	nq	nq	nq	nq	0.70	nq	nq	nq	nq	nq	nq	nq	nq	nq	0.15
9.	δ-3-Carene	1014	nq	nq	5.92	5.34	nq	nq	nq	nq	9.19	1.62	nq	17.85	9.37	nq	nq
10.	α-Terpinene	1017	nq	nq	nq	nq	nq	nq	nq	nq	nq	nq	nq	nq	nq	2.60	3.30
11.	o-Cymene	1026	nq	nq	nq	nq	nq	nq	nq	nq	nq	1.32	nq	nq	nq	nq	nq
12.	Limonene	1028	11.01	9.89	9.11	7.57	nq	6.38	0.24	0.75	2.41	2.03	5.92	30.01	32.0	2.79	4.16
13.	β-Phellandrene	1030	nq	nq	nq	nq	2.00	0.46	nq	nq	nq	nq	nq	nq	nq	nq	nq
14.	(E)-β-Ocimene	1056	nq	0.09	nq	nq	0.30	nq	0.08	nq	0.05	nq	nq	nq	nq	0.89	0.20
15.	γ-Terpinene	1060	1.58	2.59	0.66	2.59	3.80	1.03	nq	0.05	0.62	0.17	0.11		0.25	4.30	5.05
16.	cis- Sabinene hydrate	1070	nq	nq	nq	nq	nq	nq	nq	nq	nq	nq	nq	nq	nq	nq	0.97
17.	Terpinolene	1087	0.90	3.13	1.41	3.21	2.78	1.78	0.10	0.65	0.93	0.74	0.77	0.67	2.69	1.98	4.29
18.	trans- Sabinene hydrate	1098	nq	nq	nq	nq	nq	nq	nq	nq	nq	nq	nq	nq	nq	nq	0.49
19.	Linalool	1104	nq	nq	nq	nq	nq	nq	nq	nq	0.27	nq	nq	nq	nq	2.04	0.43
20.	α-Thujone	1107	nq	nq	nq	nq	nq	nq	nq	nq	nq	nq	nq	nq	nq	nq	nq
21.	β-Thujone	1115	0.32	nq	nq	nq	nq	nq	nq	nq	nq	nq	nq	nq	nq	nq	nq
22.	β-Fenchol	1120	nq	0.13	nq	0.32	nq	nq	nq	nq	nq	nq	nq	nq	nq	nq	nq
23.	cis- p-menth-2 en-1-ol	1121	nq	nq	nq	nq	nq	nq	nq	nq	nq	nq	nq	nq	nq	0.44	0.53
24.	α-Campholenal	1128	0.31	0.24	nq	0.33	nq	0.54	0.15	0.46	0.08	1.22	1.27	nq	0.03	nq	nq
25.	cis-Verbenol	1138	nq	0.35	nq	nq	nq	nq	nq	nq	nq	nq	nq	nq	nq	nq	nq
26.	trans-p-menth-2 en-1-ol	1140	nq	nq	nq	nq	nq	nq	nq	nq	nq	nq	nq	nq	nq	0.31	0.27
27.	trans- Limonene oxide	1141	nq	nq	nq	nq	nq	nq	nq	nq	nq	nq	nq	nq	0.14	nq	nq
28.	Pinocarveol	1142	nq	nq	nq	0.54	nq	0.53	0.20	0.44	nq	2.13	1.56	nq	nq	nq	nq
29.	Camphor	1145	0.41	1.43	0.35	1.10	0.44	nq	nq	nq	nq	1.02	nq	nq	nq	nq	nq
30.	trans-Verbenol	1146	nq	nq	nq	1.12	0.16	0.78	nq	nq	nq	1.14	nq	nq	nq	nq	nq
31.	Citronellal	1153	nq	nq	nq	nq	nq	nq	nq	nq	nq	nq	nq	nq	nq	0.18	nq
32.	Sabina ketone	1159	0.12	nq	nq	nq	0.37	nq	nq	nq	nq	nq	nq	nq	nq	nq	nq
33.	trans-Pinocamphone	1161	nq	nq	nq	nq	nq	nq	nq	nq	nq	0.42	nq	nq	nq	nq	nq
34.	Pinocarvone	1164	nq	nq	nq	nq	nq	nq	0.08	nq	nq	nq	0.37	nq	nq	nq	nq
35.	Borneol	1165	nq	nq	nq	0.23	nq	nq	nq	nq	nq	nq	nq	nq	0.04	nq	nq
36.	p-mentha-1,5-dien-8-ol	1168	nq	0.26	nq	0.57	nq	0.52	0.24	0.66	0.12	1.43	3.02	0.21	nq	nq	nq
37.	Terpinen-4-ol	1174	2.18	0.22	0.13	0.74	6.26	1.65	nq	0.12	0.17	0.45	0.57	0.19	0.30	6.80	7.54
38.	p-Cymen-8-ol	1186	nq	nq	nq	nq	0.18	0.13	nq	nq	nq	0.36	nq	nq	nq	nq	nq
39.	α-Terpineol	1188	0.19	0.20	nq	0.71	0.75	0.30	nq	nq	0.28	1.02	nq	0.49	0.07	0.35	0.29

40.	Myrtenal	1191	nq	nq	nq	nq	nq	nq	0.16	nq	nq	nq	nq	nq	nq		
41.	cis-Piperitol	1194	nq	nq	nq	nq	nq	nq	nq	nq	nq	nq	nq	nq	nq	0.16	0.13
42.	Myrtenol	1195	0.17	nq	nq	0.24	0.41	0.26	nq	0.12	nq	0.41	0.64	nq	nq	nq	nq
43.	Verbenone	1204	nq	nq	nq	nq	0.11	0.32	0.10	0.16	nq	0.89	1.39	nq	nq	nq	nq
44.	trans-Piperitol	1206	nq	nq	nq	nq	0.21	nq	nq	nq	nq	nq	nq	nq	nq	nq	nq
45.	trans-Carveol	1221	nq	0.17	nq	0.18	0.17	0.22	0.05	0.14	0.08	0.54	1.75	nq	nq	nq	nq
46.	α-Fenchyl acetate	1220	0.20	nq	nq	nq	nq	nq	nq	nq	nq	nq	nq	nq	nq	nq	nq
47.	p-mentha-1(7),8-dien-2-ol	1231	nq	nq	nq	nq	nq	nq	nq	nq	nq	nq	0.14	nq	nq	nq	nq
48.	cis-Carveol	1237	nq	nq	nq	nq	0.04	nq	nq	nq	nq	0.03	0.32	nq	nq	nq	nq
49.	Citronellol	1238	nq	nq	nq	nq	0.05	nq	nq	0.03	0.94	0.05	nq	nq	nq	0.68	0.09
50.	Carvone	1242	nq	nq	nq	nq	0.07	nq	nq	nq	nq	0.06	0.83	nq	nq	nq	nq
51.	Carvacrol, methyl ether	1248	nq	nq	nq	0.09	nq	nq	nq	0.12	nq	0.05	nq	nq	nq	nq	0.06
52.	Piperitone	1253	nq	nq	nq	nq	0.11	nq	nq	nq	0.09	0.33	nq	nq	nq	nq	nq
53.	Linalyl acetate	1257	nq	nq	nq	nq	nq	nq	nq	nq	1.35	nq	nq	nq	nq	0.53	0.06
54.	Methyl citronellate	1261	nq	nq	nq	nq	nq	nq	nq	nq	nq	nq	nq	nq	nq	5.48	1.63
55.	Myrtanol	1262	nq	nq	nq	nq	nq	nq	nq	nq	nq	nq	0.19	nq	nq	nq	nq
56.	Isopulegyl acetate	1271	nq	nq	nq	nq	nq	nq	nq	nq	2.40	0.11	nq	nq	nq	nq	nq
57.	(-)-Bornyl acetate	1278	0.17	0.60	0.10	0.36	0.43	0.74	nq	nq	nq	0.05	0.06	nq	nq	0.10	0.22
58.	Sabinyl acetate	1290	0.78	nq	nq	nq	nq		nq	nq	nq	nq	nq	nq	nq	0.10	0.20
59.	2-undecanone	1300	nq	nq	nq	nq	nq	0.10	nq	nq	nq	nq	nq	nq	nq	nq	nq
60.	2,4-E-Decadienol	1320	2.49	nq	1.42	nq	nq	nq	nq	nq	nq	nq	nq	nq	nq	nq	nq
61.	Methyl geranate	1324	nq	nq	nq	nq	nq	nq	nq	nq	nq	nq	nq	nq	nq	0.21	0.07
62.	δ-Elemene	1338	nq	nq	nq	nq	0.13	0.21	nq	nq	nq	0.19	nq	nq	nq	0.08	0.70
63.	α-Cubebene	1343	0.08	nq	nq	nq	0.13	0.89	0.74	0.68	nq	nq	0.17	0.99	1.07		0.14
64.	α-Terpinyl acetate	1346	nq	nq	nq	nq	nq	nq	nq	nq	2.55	1.38	nq	nq	nq	nq	nq
65.	α- Ylangene	1373	nq	nq	nq	nq	nq	nq	nq	nq	nq	nq	nq	0.06	0.14	nq	nq
66.	α-Copaene	1376	0.31	nq	0.22	nq	3.15	1.93	0.49	0.42	0.20	0.04	0.22	1.18	1.10	0.42	0.91
67.	β-Bourbonene	1381	nq	nq	nq	nq	0.36	nq	0.68	nq	0.37	nq	0.39	0.16	nq	nq	nq
68.	β-Cubebene	1385	nq	nq	nq	nq	nq	nq	0.72	nq	nq	nq	nq	0.13	0.19	nq	nq
69.	β-Elemene	1389	nq	0.19		0.17	nq	3.83	0.76	nq	1.25	0.66	0.10	0.17	nq	0.16	0.61
70.	7-epi-Sesquithujene	1392	0.37	nq	0.40	nq	nq	nq	nq	nq	nq	nq	nq	nq	nq	nq	nq
71.	Longifolene	1406	nq	nq	nq	nq	nq	nq	nq	0.21	nq	nq	nq	nq	nq	nq	nq
72.	Z-Caryophyllene	1410	nq	nq	nq	nq	nq	nq	nq	nq	nq	nq	0.22	nq	nq	nq	nq
73.	β-Funebrene	1414	3.25	2.85	5.33	2.61	2.88	nq	1.45	1.60	nq	nq	nq	nq	nq	nq	nq
74.	b-Caryophyllene	1419	1.87	2.51	2.86	1.48	2.39	1.21	3.37	2.73	5.42	1.97	6.02	1.75	1.57	0.27	0.68
75.	β-Copaene	1430	nq	nq	nq	nq	nq	nq	nq	nq	0.38	nq	nq	0.35	0.42	nq	nq
76.	Widdrene (thujopsene)	1431	0.91	0.70	1.46	0.59			6.52	2.72	nq	nq	nq	nq	nq	nq	nq
77.	γ-Elemene	1436	nq	nq	nq	nq	1.63	3.91	nq	nq	0.49	1.75	nq	nq	nq	0.21	1.45
78.	cis-Muurola-3,5-diene	1446	nq	nq	nq	nq	nq	nq	1.65	nq	nq	nq	nq	nq	nq	0.07	nq
79.	α-Humulene	1447	1.94	2.78	2.47	1.38	2.25	1.55	1.36	2.76	3.50	1.21	4.61	1.58	1.40	0.13	0.10
80.	trans-Muurola-3,5-diene	1453	nq	nq	nq	nq	nq	nq	nq	nq	nq	nq	nq	0.16		0.07	nq
81.	Bicyclosesquiphellandrene	1454	nq	nq	nq	nq	nq	nq	nq	nq	nq	nq	nq	0.74	0.24	0.17	nq
82.	Thujopsadiene	1455	nq	nq	nq	nq	nq	nq	0.31	nq	nq	nq	nq	nq	nq	nq	nq
83.	E-β-Farnesene	1462	0.92	nq	nq	nq	nq	0.42	nq	nq	nq	0.06	0.10	nq	nq	0.04	0.09
84.	α-Acoradiene	1463	nq	0.09	nq	0.13	nq	nq	nq	nq	nq	nq	nq	nq	nq	nq	nq
85.	cis-Cadina-1(6),4-diene	1463	1.06	0.27	1.18	0.17	0.12	0.24	1.19	nq	nq	nq	nq	nq	nq	nq	nq
86.	cis-Muurola-4(14),5-diene	1465	nq	nq	nq	nq	nq	nq	nq	nq	0.23	nq	nq	nq	0.2	nq	nq
87.	β-Acoradiene	1469	nq	0.37	0.87	0.35	nq	nq	nq	nq	nq	nq	nq	nq	nq	nq	nq
88.	Dauca-5,8-diene	1472	nq	nq	nq	nq	nq	nq	nq	nq	nq	nq	0.20	nq	nq	nq	nq

89.	trans- Muurola-4(14),5-diene	1488	nq	nq	nq	nq	nq	nq	nq	nq	nq	nq	nq	nq	0.10	0.19	nq
90.	trans-Cadina-1(6),4-diene	1471	nq	nq	nq	nq	nq	nq	nq	nq	nq	nq	nq	nq	nq	0.12	0.07
91.	γ-Muurolene	1478	nq	nq	nq	0.18	nq	nq	0.77	nq	nq	nq	0.61			0.23	nq
92.	Germacrene d	1481	1.75	1.81	1.33	0.32	5.15	6.18	5.41	8.77	12.34	2.95	0.39	9.76	10.05	0.57	1.01
93.	ar-Curcumene	1485	0.08	nq	0.11	nq	nq	nq	nq	nq	nq	nq	nq	3.51	nq	nq	nq
94.	β-Selinene	1487	nq	0.14	nq	0.12	0.38	0.50	nq	0.18	0.22	0.15	0.22	nq	0.09	nq	nq
95.	epi-Bicyclo sesquiphellandrene	1488	1.00	0.06	0.75	nq	0.13	0.23	1.48	1.23	0.14	nq	0.34	nq	nq	nq	nq
96.	Valencene	1491	nq	nq	nq	nq	nq	nq	nq	nq	nq	nq	0.31	nq	nq	nq	nq
97.	α-Selinene	1493	nq	0.11	nq	0.11	nq	nq	nq	nq	nq	nq	nq	nq	nq	nq	nq
98.	epi-Cubebol	1494	nq	nq	nq	nq	nq	nq	2.00	nq	nq	nq	nq	nq	nq	0.30	0.15
99.	γ-Amorphene	1495	nq	nq	nq	nq	nq	nq	nq	nq	nq	nq	nq	nq	0.68	nq	nq
100.	β-Alaskene	1496	0.89	0.54	1.28	0.40	nq	nq	nq	nq	nq	nq	nq	0.97	nq	nq	nq
101.	Viridiflorene	1498	nq	nq	nq	nq	nq	nq	nq	nq	0.80	0.22	nq	nq	nq	nq	nq
102.	Bicyclogermacrene	1500	nq	nq	nq	nq	1.32	1.97	nq	nq	nq	nq	nq	nq	nq	nq	nq
103.	α-Cuprenene	1503	nq	nq	0.26	nq	nq	nq	nq	nq	nq	nq	nq	nq	nq	nq	nq
104.	α-Muurolene	1504	0.54	0.51	1.01	nq	0.60	0.73	10.56	6.38	0.74	0.32	2.62	0.72	0.46	0.68	0.47
105.	β-Himachalene	1507	nq	0.13	nq	0.10	nq	nq	0.80	nq	nq	nq	nq	nq	nq	nq	nq
106.	α-Alaskene	1511	1.74	0.86	1.80	0.91	nq	nq	nq	nq	nq	nq	nq	nq	nq	nq	nq
107.	δ-Amorphene	1514	nq	nq	nq	nq	nq	0.12	nq	0.13	0.15	0.04	nq	nq	0.22	nq	nq
108.	Cubebol	1515	nq	nq	0.45	nq	nq	nq	nq	nq	nq	nq	nq	nq	nq	nq	nq
109.	trans-Calamenene	1522	nq	nq	nq	nq	nq	nq	0.55	nq	nq	nq	nq	0.35	nq	nq	nq
110.	δ-Cadinene	1524	2.64	0.56	1.42	0.21	4.50	3.90	8.39	6.83	2.18	0.76	1.24	1.55	2.04	3.26	2.08
111.	γ-Cadinene	1525	nq	nq	nq	nq	0.70	0.92	3.12	2.62	0.95	0.22	1.10	3.63	1.71	0.95	nq
112.	β-Sesquiphellandrene	1526	nq	nq	nq	0.07	nq	nq	nq	nq	nq	nq	nq	nq	nq	nq	nq
113.	(E)-γ-Bisabolene	1527	0.40	0.19	0.49	0.17	nq	nq	nq	nq	nq	nq	nq	nq	nq	nq	nq
114.	trans-Cadina-1,4-diene	1529	0.39	nq	0.46	nq	0.12	0.25	1.04	0.80	0.22	nq	0.10	nq	0.13	0.09	0.06
115.	10-epi-Cubebol	1535	nq	nq	nq	nq	nq	nq	nq	0.62	nq	nq	nq	nq	nq	nq	nq
116.	α-Cadinene	1539	0.10	nq	nq	nq	nq	nq	nq	0.21	0.17	nq	0.08	0.21	0.18	0.34	0.10
117.	α-Calacorene	1542	nq	nq	nq	nq	0.14	nq	0.48	0.34	nq	nq	0.28	0.07	nq	nq	nq
118.	Elemol	1549	nq	nq	nq	nq	nq	nq	nq	nq	0.25	0.87	nq	nq	nq	0.66	0.99
119.	β-Calacorene	1561	nq	nq	nq	nq	0.05	nq	nq	0.18	nq	nq	nq	0.07	nq	nq	nq
120.	Germacrene B	1564	nq	nq	nq	nq	2.18	5.13	nq	nq	0.90	1.92	nq	nq	nq	0.26	1.57
121.	Nerolidol	1571	nq	nq	nq	nq	0.40	0.25	nq	nq	0.21	0.18	nq	nq	nq	nq	nq
122.	E-Nerolidol	1572	nq	nq	nq	nq	nq	nq	nq	nq	nq	nq	nq	nq	nq	nq	0.08
123.	Germacrene d-4-ol	1575	nq	nq	nq	nq	nq	1.21	nq	nq	0.25	nq	nq	nq	nq	2.92	1.03
124.	Spathulenol	1577	nq	nq	nq	nq	1.68	0.95	nq	nq	nq	nq	nq	nq	nq	nq	nq
125.	Caryophyllene oxide	1582	nq	nq	nq	nq	0.31	0.22	1.83	1.28	1.00	1.32	13.62	0.60	0.05	nq	nq
126.	allo-Cedrol	1589	1.53	2.12	2.51	1.97	0.17	0.27	0.45	0.49	0.26	0.40	0.48	nq	nq	nq	nq
127.	Salvial-4(14)-en-1-one	1594	nq	nq	nq	nq	nq	nq	nq	nq	0.15	nq	nq	nq	0.27	nq	nq
128.	α-Cedrol	1601	22.40	19.46	27.73	17.79	0.20	0.17	12.80	14.12	0.10	0.15	0.80	nq	nq	nq	nq
129.	Humulene epoxide ii	1602	nq	0.14	nq	0.15	0.50	0.36	nq	0.54	0.70	0.71	7.33	0.66	0.04	nq	nq
130.	β-Oplopenone	1607	nq	nq	nq	nq	nq	nq	nq	nq	nq	nq	nq	nq	nq	1.39	0.17
131.	1,10-di-epi-Cubenol	1610	nq	nq	nq	nq	0.08	0.23	nq	nq	nq	nq	nq	nq	nq	0.09	0.05
132.	1-epi-Cubenol	1623	0.94	nq	0.99	nq	0.26	0.22	3.98	2.39	0.13		0.75		0.04	0.14	0.08
133.	α-Acorenol	1627	nq	0.24	nq	0.19	nq	nq	nq	nq	nq	nq	nq	nq	nq	nq	nq
134.	γ-Eudesmol	1630	nq	nq	nq	nq	nq	0.08	nq	nq	nq	nq	nq	nq	nq	nq	0.08
135.	β-Acorenol	1637	0.24	0.22	0.40	0.17	nq	nq	nq	nq	nq	nq	nq	nq	nq	nq	nq

136.	epi-α-Cadinol	1638	0.95	0.11	nq	nq	0.35	nq	nq	nq	0.31	nq	0.48	0.99	0.37	nq	nq
137.	epi-α- Murrolol	1641	nq	nq	0.32	nq	0.87	0.62	nq	0.67	0.22	nq	nq	nq	nq	1.92	0.68
138.	δ-Cadinol	1646	0.18	nq	nq	nq	nq	nq	0.93	0.84	nq	nq	0.44	nq	0.07	0.33	0.16
139.	α-Eudesmol	1653	nq	nq	nq	nq	nq	nq	nq	nq	nq	nq	nq	nq	nq	nq	0.08
140.	α-Cadinol	1656	0.93	nq	nq	nq	1.23	1.32	nq	0.77	0.43	nq	1.01	0.58	0.18	2.66	0.80
141.	Germacra-4(15),5,10(14)-trien-1-α-ol	1681	nq	nq	nq	nq	0.19	0.14	0.80	nq	0.59	nq	0.25	nq	nq	nq	nq
142.	Shyobunol	1684	nq	nq	nq	nq	0.26	0.39	nq	nq	0.08	nq	nq	nq	nq	0.13	0.33
143.	2,3-Dihydro-farnesol	1697	nq	nq	nq	nq	nq		nq	nq	1.37	nq	nq	nq	nq	0.12	nq
144.	2,6-Farnesol	1720	nq	nq	nq	nq	0.04	0.08	nq	nq	nq	nq	nq	0.99	nq	0.09	nq
145.	2,6-Farnesal	1743	nq	nq	nq	nq	nq	nq	nq	nq	0.05	nq	nq	nq	nq	0.32	nq
146.	14-oxy-α-muurolene	1768	nq	nq	nq	nq	nq	nq	nq	0.04	nq	nq	nq	nq	nq	nq	nq
147.	14-hydroxy-α-muurolene	1780	nq	nq	nq	nq	nq	nq	nq	0.05	nq	nq	nq	nq	nq	nq	nq
148.	β-Bisabolenol	1789	nq	nq	0.07	nq	nq	nq	nq	nq	nq	nq	nq	nq	nq	nq	nq
149.	Pimaradiene	1949	nq	nq	0.08	nq	0.29	nq	nq	0.54	0.08	nq	0.22	nq	nq	nq	nq
150.	Sandaracopimara-8(14),15-diene	1969	nq	nq	nq	nq	nq	nq	nq	nq	nq	nq	nq	nq	0.03	nq	nq
151.	Manool oxide	1987	0.05	0.27	nq	nq	nq	nq	nq	1.44	nq	nq	1.03	1.08	nq	nq	nq
152.	13-epi-manool oxide	2011	0.09	nq	nq	nq	nq	nq	3.55	0.03	0.22	0.12	nq	nq	0.05	nq	nq
153.	Abieta-8,12-diene	2015	nq	0.35	0.07	nq	nq	nq	nq	0.07	0.06	0.05	nq	nq	nq	0.13	nq
154.	Abietatriene	2056	nq	nq	0.09	nq	nq	nq	0.39	0.18	0.07	nq	2.03	0.45	0.43	0.03	0.02
155.	Abietadiene	2087	0.16	0.70	0.57	nq	0.09	nq	0.49	0.06	0.27	0.31	1.65	0.04	0.05	0.79	0.58
156.	Abieta-8(14),13(15)-diene	2164	nq	0.11	nq	nq	nq	nq	nq	nq	nq	0.03	nq	nq	nq	0.03	nq
157.	Sandaracopimarinal	2192	nq	0.06	nq	nq	nq	0.38	nq	1.30	0.08	0.03	0.12	0.08	0.03	nq	nq
158.	Sandaracopimarinol	2269	nq	nq	nq	nq	nq	nq	nq	0.28	nq	nq	nq	nq	nq	0.01	nq
159.	4-epi-abietal	2298	0.13	0.33	0.10	nq	nq	nq	nq	nq	0.36	0.52	nq	nq	nq	nq	0.06
160.	Isopimarol	2310	nq	nq	nq	nq	nq	nq	nq	0.04	nq	nq	nq	nq	nq	0.15	
161.	Abieta-7,13-dien-3-one	2313	nq	0.17	nq	nq	nq	nq	nq	nq	0.07	0.07	nq	nq	nq	0.15	0.04
162.	Totarol	2314	nq	nq	nq	nq	nq	nq	nq	nq	nq	nq	nq	0.08	0.07	nq	nq
163.	Trans-ferruginol	2332	nq	nq	nq	nq	nq	nq	nq	nq	nq	nq	nq	nq	0.02	nq	nq
164.	Abietol	2401	nq	nq	nq	nq	nq	nq	nq	nq	nq	0.03	nq	nq	nq	nq	nq
			**48**	**48**	**41**	**43**	**59**	**54**	**45**	**53**	**64**	**60**	**54**	**39**	**45**	**61**	**56**

Je= Juniperus excelsa, Jf= Juniperus foetidissima, Jcc= Juniperus communis subsp. *communis, Jm= Juniperus macrocarpa, Jt= Juniperus turbinate, Jod= Juniperus oxycedrus* subsp. *deltoides, Jd= Juniperus drupacea, Js= Juniperus sabina;* L= leaves, C= cones, Aerial = leaves and cones were not distinguished, KI= Kovats Index, nq= not quantified.
